# Supplementary material for: SyNDI: synchronous network data integration framework
Source: BMC Bioinformatics. 2018 Nov 6;19:403. doi: 10.1186/s12859-018-2426-5 (PMC6219086; doi:10.1186/s12859-018-2426-5)
Supplement: Supplementary file 5 — Files of the example presented in Additional file 4. This additional file is a zip package that contains all files used in Additional file 4. Detailed descriptions of each of these files are presented in a README file included in this zip package. (GZ 30411 kb) [file 12859_2018_2426_MOESM5_ESM.gz › SyNDITools/Meme2Fimo/meme_4.9.0_4.tar.gz!/meme_4.9.0/website/cgi-bin/dreme.tmpl]

DREME - Submission form


Select a file of FASTA formatted
DNA sequences or paste in actual FASTA formatted DNA sequences to search for small regular expression
motifs.

DREME works best with sequences which are less than 500 nucleotides
long so if you have very long sequences DREME might work better if you
subdivide them. For ChIP-seq we recommend using sequences of length 100
centered around the peak. The more sequences that you can give DREME the
more subtle the motifs it can find.

See the sample sequences
to get an idea of input that works well for DREME.

[ close ]

The email is used to notify you of your results.

[ close ]

The job description will be included in the notification email you
receive and in the DREME output.

[ close ]

By default DREME will perform a dinucleotide shuffle on
the input sequences and use them as the comparison but with
this option custom comparative sequences can be specified.

[ close ]

Input comparative sequences which have approximately the same length
distribution and background frequencies but which are unlikely to contain
the motifs that you are attempting to find.

Note that using comparative sequences that are much longer or shorter
than your input sequences has the potential to create incorrect
p-values.

[ close ]

The expected number of false positives. This may be any positive
number.

[ close ]

The maximum number of motifs to find.

[ close ]

|  |  |
| --- | --- |
|  | MEME Suite  The MEME Suite web application requires the use of JavaScript  Javascript doesn't seem to be available on your browser.  Version  Use this form to submit DNA sequences to DREME. DREME will analyze your sequences for repeated patterns that are enriched when compared to a discriminative set and produce a description (motif) for each pattern it discovers. For more information about DREME, see this Tutorial .  Data Submission Form Discover short regular expression motifs.  Input the sequences  Upload DNA sequences in which you want to find motifs  or paste the sequences  Input job queue details  Enter your email address.  Re-enter your email address.     Optionally enter a job description.  Advanced Options  Advanced Options  Select comparison source  Shuffled sequences  Custom     Input the comparative sequences  or paste the sequences    Treatment of reverse complement strands  Use given strand only  Set the limits  •  *E*-value ≤ |
